# Pulsed-laser micropatterned quantum-dot array for white light source

**DOI:** 10.1038/srep23563

**Published:** 2016-03-23

**Authors:** Sheng-Wen Wang, Huang-Yu Lin, Chien-Chung Lin, Tsung Sheng Kao, Kuo-Ju Chen, Hau-Vei Han, Jie-Ru Li, Po-Tsung Lee, Huang-Ming Chen, Ming-Hui Hong, Hao-Chung Kuo

**Affiliations:** 1 Department of Photonics & Institute of Electro-Optical Engineering, National Chiao Tung University, Hsinchu 30010, Taiwan; 2 Institute of Photonic System, National Chiao Tung University, Tainan 711, Taiwan; 3 Department of Electrical and Computer Engineering, National University of Singapore, 4 Engineering Drive 3, 117576 Singapore, Singapore

## Abstract

In this study, a novel photoluminescent quantum dots device with laser-processed microscale patterns has been demonstrated to be used as a white light emitting source. The pulsed laser ablation technique was employed to directly fabricate microscale square holes with nano-ripple structures onto the sapphire substrate of a flip-chip blue light-emitting diode, confining sprayed quantum dots into well-defined areas and eliminating the coffee ring effect. The electroluminescence characterizations showed that the white light emission from the developed photoluminescent quantum-dot light-emitting diode exhibits stable emission at different driving currents. With a flexibility of controlling the quantum dots proportions in the patterned square holes, our developed white-light emitting source not only can be employed in the display applications with color triangle enlarged by 47% compared with the NTSC standard, but also provide the great potential in future lighting industry with the correlated color temperature continuously changed in a wide range.

Recently the light-emitting diode (LED) with white-light emission has become a key component in optoelectronic applications, especially for the low power-consumption lighting devices and full-color displays. Currently, the most common method for the white-light LED fabrication in industrials is the use of the gallium nitride (GaN) based blue LEDs to pump yellow phosphors^1^,^2^. Phosphor-based white-light LEDs provide the high efficiency light emission performance and are usually employed as the backlight source in displays. This approach is efficient and cost effective. Although the correlated color temperature (CCT) of the light emitted from phosphor-based white-light LEDs can be continuously tuned in a wide range with different phosphor doping concentrations, their color gamut is still limited and cannot reproduce the natural colors^3^,^4^,^5^. To enlarge the color gamut, many new phosphor materials with narrow emission bandwidth have been developed and can be mixed together to render the natural white-light emission^6^,^7^.

Colloidal quantum dots (QDs), because of their significant features such as the narrow emission bandwidth, broad absorption spectrum and tunable size quantization effect, have been extensively developed and applied for the display applications^8^. For example, Chen *et al*. used the spray coating method to separate the different color-emissions QDs on a 2-inch glass substrate, generating a large homogeneous white light emission plate as a backlight^9^. Recently, an alternative technique for deploying QDs which is called the quantum dot enhancement film (QDEF) provides a straightforward application in the integration with the existing display manufacturing processes. The amount and ratio of QDs with different color emissions determine the final color specification^10^. However, the mixture of different quantum-dots may cause significantly reabsorption effect, leading to the CIE change and the efficiency reduction of constitute QDs^11^,^12^,^13^.

In this paper, we exploit the pulsed laser direct writing and aerosol jet (AJ) spray coating techniques to develop a novel photoluminescent quantum dots device with microscale patterns for the uses as a white light emitting source^14^. The pulsed laser ablation technique^15^,^16^,^17^ was employed to directly fabricate microscale square holes with nano-ripple structures onto the sapphire substrate of a flip-chip blue light-emitting diode, confining sprayed quantum dots into well-defined areas and eliminating the coffee ring effect. The AJ spray coating method is a mask-free technique and can be used to reduce the volume of QD films, decrease the consumption of QDs, and easily deposit QDs on target areas with determined QDs proportions^18^. With these two advanced techniques, QDs of different color emissions can be sprayed at specific areas with proportional compositions and separated to prevent the interactions between each other. With a flexibility of controlling the quantum dots proportions in the patterned square holes, our developed white-light emitting source not only can be employed in the display applications, but also provide the great potential in future illumination industry.

## Results and Discussion

**Figure 1:**
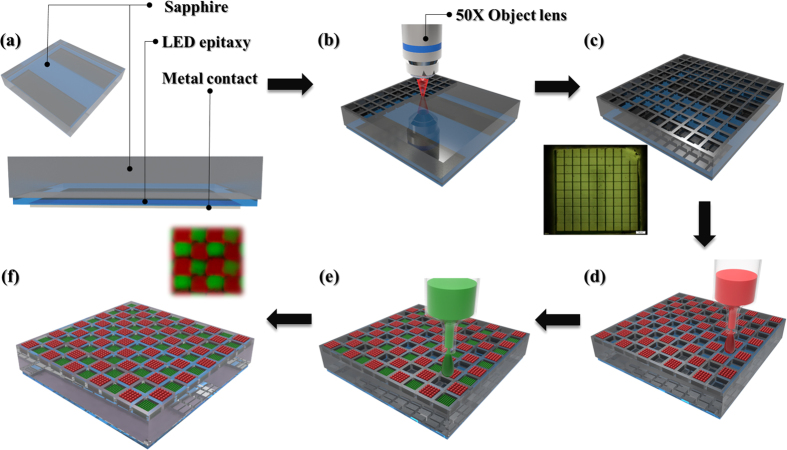
Preparation of the QD white-light LEDs. (**a**) Schematic diagram of the flip-chip blue LED structure. (**b**) Direct structural patterning on the sapphire substrate through the laser ablation using an 800-nm pulsed *fs* laser beam focused on the sapphire surface. (**c**) Patterned pixel structure. The pixel size is in a square area of 40 μm × 40 μm. The inset shows the OM image of the fabricated sample. Via the AJ spray coating method, QDs of green and red light emission can be sequentially injected into the square holes as the process shown in (**d**,**e**). (**f**) The QD white-light LED device. The inset presents the fluorescence microscope image of the chessboard patterned surface with QDs of red and green emission.

In the process of fabricating the developed white-light LED, a flip-chip blue LED was prepared to be used as a light emitting source to irradiate the sprayed ZnS and CdSe QDs for green and red light emission, respectively. The chip was in a square dimension of 510 μm × 510 μm, and the emission wavelength was approximately at 450 nm as the chip operated at a driving current of 50 mA. This LED structure is schematically shown in Fig. 1a, where the LED epitaxial layer sandwiched between a top sapphire substrate and a bottom metal contact. To pattern an array of microscale square holes on the sapphire substrate, as shown in Fig. 1b, an 800 nm Ti:Sapphire femtosecond (*fs*) laser (Clark-MXR, CPA-2010) was employed to directly ablate the sapphire material, while the pulse duration and the repetition rate of the *fs* laser beam were controlled at 100 *fs* and 1 kHz, respectively. Through a 50X objective lens, the incident laser light beam can be concentrated to a focused spot at the size around 1.5 μm in diameter. The laser-processing area can be well defined using a three-dimensional translation stage. Unlike other lithography techniques and etching facilities, the laser ablation method not only provides the fine structure patterning process but also can be directly employed in atmospheric environment without any pre-treatments on samples or the demands of a high vacuum exhausting system. Regarding the precision of pattern fabrication, the smallest linewidth on the sapphire material is around 1.5 μm in the laser-processing system. Such a fabrication precision fulfills the requirement in patterning our samples with minimum separation of QDs emitted in different colors. Figure 1c shows the schematic laser-ablated pattern of a microscale square holes array on the sapphire substrate in a flip-chip blue LED structure, while the inset displays the top-view optical microscope (OM) image of the fabricated 10 × 10 square holes array. The laser-ablated hole is 40 μm × 40 μm in a square dimension and 5 μm in depth. The square holes are separated by sidewall boundaries of 10 μm in width.

After completing the laser ablation process, the AJ printing technology was employed to spray QDs with different color emissions into individual square microholes, preventing the unrequired QDs mixture in our developed QD white-light LEDs. The AJ printing equipment was developed by the Optomec Inc. The QDs can be homogenous sprayed onto the sample surface by aerosolizing the QD droplets and precisely injected into specific locations by manipulating the dynamic of surrounding air^11^. To start with the AJ spray coating of QDs, first the QDs with red (green) light emission were prepared in toluene solution with the concentration of approximately 5 mg/mL. Then, 1 cm^3^ of the prepared QD solution was loaded into an ultrasonic atomizer and ejected as a gas stream. The AJ spray performance can be adjusted by controlling the distance between the deposition head, pneumatic atomization frequency, carrier gas flow rate, and the amount of focusing gas input. The linewidth of the sprayed QDs in a line can be achieved at around 40 μm. Via the control between the rate of an on/off shutter and moving speed of the translation table underneath, QDs can be included in a single droplet format and sprayed individually into the fabricated microsholes. The QDs droplet is around at 30 μm in diameter. In our work, we alternately sprayed QDs with red and green light emission in a chessboard pattern as described in the schematic images shown in Fig. 1d,e. Twice the number of QDs droplets with green light emission were sprayed into the microholes due to the higher down-conversion efficiency of the QDs in red light emission. Finally, a white-light LED based on the combination of a flip-chip blue LED and QDs of red and green light emission in a patterned holes array was produced. Figure 1f shows the schematic diagram and the inset indicates the fluorescence microscope image of QDs sprayed white-light LED chip.

**Figure 2:**
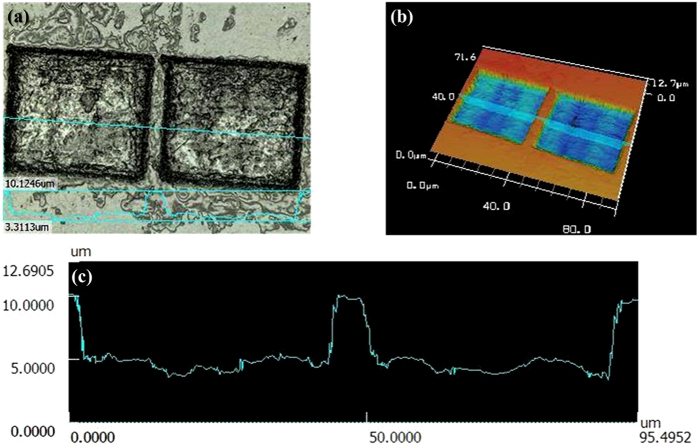
Morphology characterization of the laser-ablated microholes. The morphology of the laser-ablated square holes is characterized by using a laser scanning confocal microscope and the results are shown in (**a**,**b**). (**c**) Indicates the extracted surface profile of the ablated holes. The length and the depth of the ablated square holes are around 40 μm and 5 μm, respectively.

**Figure 3:**
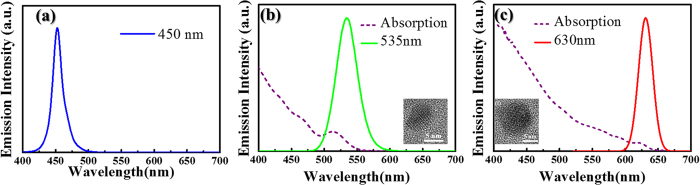
Spectral characterizations. (**a**) The flip-chip blue LED exhibits light emission at the central wavelength of 450 nm. (**b**,**c**) Show the absorption (dashed line) and PL emission (solid line) spectra of the ZnS and CdSe QDs. The ZnS QDs in toluene solution show the light emission at a peak wavelength of 535 nm, while the central emission wavelength of the CdSe QDs is at 630 nm. The insets in (**b**,**c**) show the TEM images of the QDs used in this study.

The morphology of the laser-processed square holes was characterized using a laser scanning confocal microscope and the result is shown in Fig. 2. According to the image, the length and the depth of the ablated square holes are around 40 μm and 5 μm, respectively. The width of the sidewall boundary is around 10 μm. With the laser-ablated structure, the light emitting from the blue light-emitting chip still exhibits the light emission at a central emission wavelength of 450 nm with a bandwidth of 40 nm, and the light intensity remains a uniform distribution at a wide angular range. The optical properties of the sprayed ZnS and CdSe QDs employed in the developed white-light LEDs were characterized by the absorption and the PL spectral measurements as the results shown in Fig. 3b,c. The ZnS QDs in toluene solution show the light emission at a peak wavelength of 535 nm, while the central emission wavelength of the CdSe QDs is at 630 nm. The TEM images as the insets in Fig. 3b,c show that the corresponding sizes of the ZnS and CdSe QDs are 7 nm and 10 nm, respectively.

**Figure 4:**
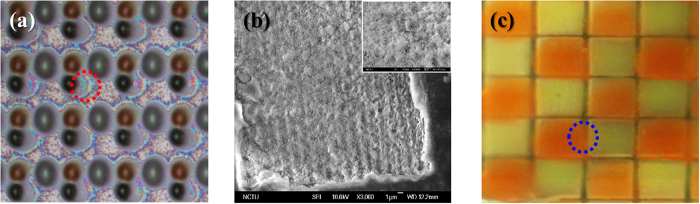
Comparison of QDs sprayed on flat and patterned sapphire substrates. (**a**) An OM image of QDs sprayed onto the flat sapphire substrate. The coffee ring effect can be clearly observed at the surrounding edge of the QD droplets. (**b**) A scanning electron microscope (SEM) image of a laser-ablated square hole. Nano-ripples structure is formed at the laser-ablated locations, providing the hydrophobic surface. With the nano-ripples structure, the sprayed QDs are well confined in the patterned area as shown in (**c**).

Figure 4a shows the OM image of the QDs sprayed on the flat sapphire surface. A significant coffee ring effect occurs because of most QDs accumulated at the surrounding edge of the QDs droplets. Compared with the QDs sprayed onto the patterned sapphire structure, the unwanted coffee ring effect can be eliminated. According to the optical microscope images of the QDs dropping onto the laser-ablated area, quite a few of the injected QDs may mix together on the boundary as the dashed circle shown in Fig. 4c. The QD mixture may result from the system vibration or the over-deposition. Compared to the QDs directly sprayed onto a flat surface as displayed in Fig. 4a, the developed technique demonstrated in this work indeed shows its advantage of separating QDs in individual locations. Also the corresponding SEM image (in the supplementary information as Fig. S1), most of the QDs are observed to accumulate at the center of the microholes, which may result from the generation of the nano-ripple structure after the laser ablation process. Such a nano-rippled surface exhibits hydrophobic characteristics and can be used to concentrate the droplets on the target area with a larger contact angle^19^,^20^,^21^,^22^,^23^. In summary, the droplets may be concentrated at the center and thus most QDs are accumulated at specific areas, as shown in Fig. 4c. The coffee ring effect is eliminated by the fabricated nano-ripple structures from the laser-ablation process.

**Figure 5:**
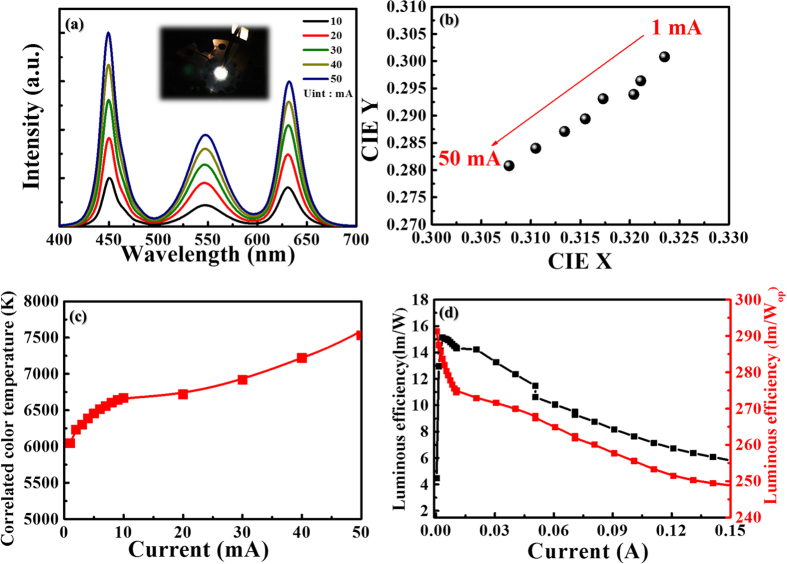
EL characterizations and color performance. (**a**) Shows the relative EL emission spectra of the QD-based white-light LEDs at driving current changed from 10 mA to 50 mA. The inset OM image shows white light emission from the developed light-emitting device. (**b**) Indicates the trace of light illumination from the photoluminescent QD-based white-light LEDs in the CIE1931 chromaticity diagram. (**c**) Shows the CCT values of the developed device operated at different driving currents. (**d**) The luminous efficiency and the luminous efficacy of radiation (LER) of the developed QD-based white light LEDs. The driving current is changed from 1 mA to 150 mA, while the LER is around 262 l_m_/W_op_ at 50 mA.

The light-emitting performance of the QD-based white-light LEDs was characterized by conducting a series of the EL spectral measurements at different driving currents. Figure 5a shows the normalized EL spectra of the developed light-emitting devices, collected at the normal direction with the current changed in range from 1 mA to 50 mA. These results can be extracted and represented with corresponding the Commission Internationale d’Eclairage (CIE) coordinates and correlated color temperature (CCT) as shown in Fig. 5b,c, respectively. With an increase of the driving current, the CIE coordinate is shifted from (0.325, 0.300) to (0.308, 0.280) and the CCT is varied from 6000 K to 7500 K. The reason for this could be attributed to the continuous increase in the amount of blue photons with the current whereas the QD emission is saturated at high currents. In order to improve the color property variation, a distributed Bragg reflector (DBR) is employed to adjoin with the photoluminescent QD-based device to suppress the number of the blue-light-emission photons. The DBR exhibits a high reflective band centered at 450 nm as the reflectance spectrum (in the supplementary information as Fig. S5a). With this added DBR structure, the ratio between the blue, green and red light emission can be kept at a constant value as the spectra displayed (in the supplementary information as Fig. S5b). The driving current is varied from 10 mA to 50 mA. Therefore, the corresponding CIE coordinates and CCT at different driving currents can be maintained at almost the same value, performing constant color properties as demonstrated (in the supplementary information as Fig. S5c,d). A DBR structure is suggested to be used to enhance the quality of light emission at different driving currents^24^. Also we found that the color shift at different viewing angles. The angle-dependent EL spectral measurements at different viewing angles (in the supplementary information as Fig. S2a). The intensity ratios of red and green light emission would be reduced with an increase of the viewing angle, which may result from most of the QDs were only dropped on the top surface of the flip-chip LED (in the supplementary information as Fig. S2b), the simulation also have similar results (Fig. S3). Since the blue light emission of the LED chip employed in this work is uniform at different angles, there is still a large amount of emitted light can be detected at a wider angle, particularly the off-axis direction. Regarding the commercialized products, usually a reflector cup would be added to LEDs to modify the far-field pattern, performing the lambertian distribution. Therefore, the color shift would be small at different viewing angles. Also, we have compared with liquid type QD solution and QD droplets, and the QD droplets is the reduction in quantum efficiency due to the solvent is dried up (Fig. S4). Figure 5d shows the measured luminous efficiency and luminous efficacy of radiation (LER) for the developed QD-based white light LED operated at different driving currents in a range from 1 mA to 150 mA. The LER of the QD white LED device is approximately 262 lm/W_op_ at 50 mA.

**Figure 6:**
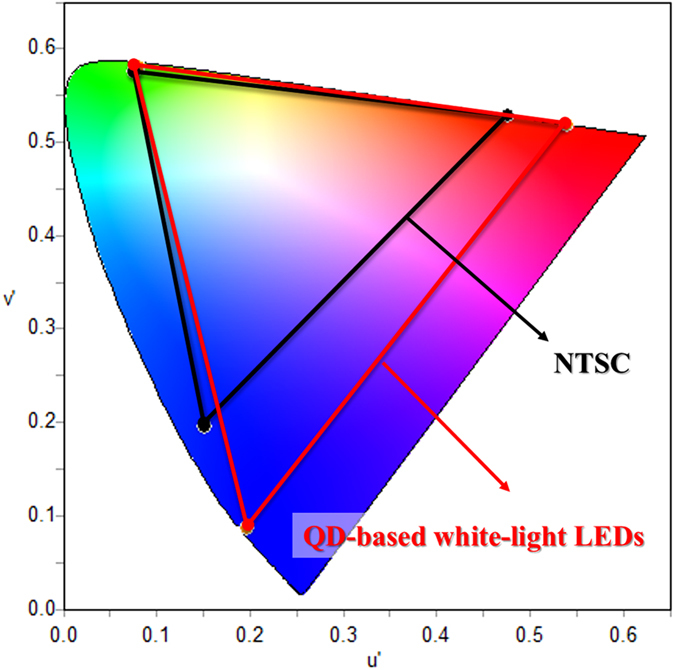
Comparison of the color triangles between the standard NTSC format and the light emission performance of the QD-based white-light LEDs in the CIE1976 color space chromaticity diagram.

**Figure 7:**
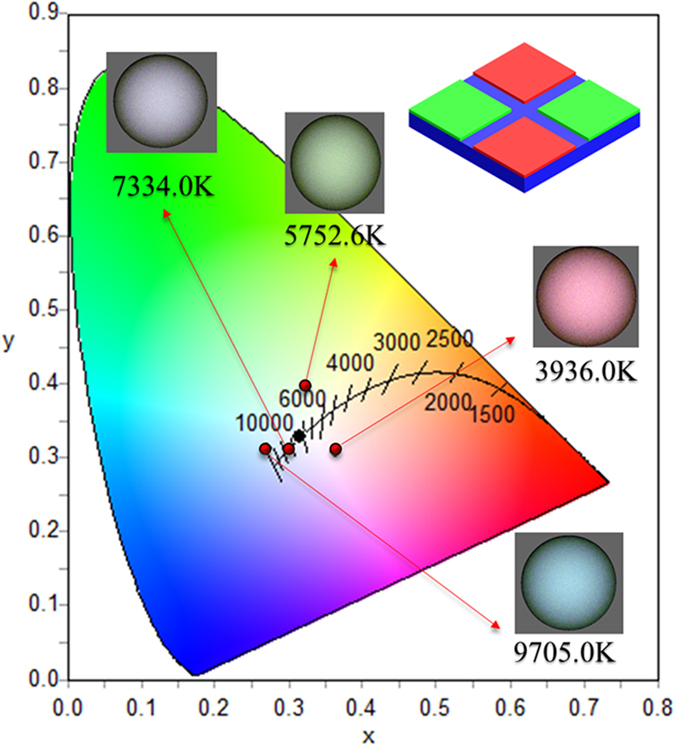
CIE simulations. Simulated CIE values for light emission from a unit pixel pattern with different proportions of red- and green-light emission QDs and blue light intensities. The values were obtained using the blackbody radiation law in OptisWorks. The inset shows the model of a unit pixel pattern and the color emission at different correlated color temperatures.

To employ the developed white-light LEDs in further display applications, we compared the CIE coordinates of our white-light LEDs with the National Television System Committee (NTSC) standard color triangle. The RGB color coordinates of the QD-based white-light LEDs are given at (0.7007, 0.2992), (0.2295, 0.7250), and (0.1500, 0.0295) respectively as represented in Fig. 6. The maximum color gamut of using the QDs white-light LEDs is greater than the standard NTSC color gamut by 47%. This result can be attributed to the narrow bandwidth of the QDs emission, giving the promising potential in displays with high color purity. Furthermore, in our developed QDs white-light LEDs, we can control the QD proportions in individual laser-ablated square holes to manipulate the CCT performance in a wide range from 3936 K to 9705 K. Figure 7 demonstrates the simulation results of the CCT performance with different proportions of QDs in green and red light emission using the OptisWorks, while the corresponding recipes of QDs proportions for different color temperatures can be obtained as shown in Table 1. Thus, our developed QDs white-light LEDs not only can be employed in the display applications, but also provide the great potential in future illumination industry.

## Conclusion

QD-based white-light LEDs with a microscale square holes array have been successfully demonstrated for white light emission by employing the pulsed laser ablation and the AJ printing techniques. After the laser treatment, the bottom of the microholes formed a nano-rippled surface with hydrophobic characteristics, and the coffee ring effect was eliminated because the QD droplets were concentrated in a specific area. The EL results, shows that the QD WLEDs exhibited stable emission upon being driven by different currents. The CCT of the QD WLEDs shifted from 6000 K to 7500 K when the current increased from 1 mA to 50 mA because of the saturation of the emission intensity of the QDs with the increase in driving current. In addition to the illumination applications, the maximum color gamut of using our QD-based white-light LEDs is enlarged and greater than the standard NTSC color gamut by 47%. In conclusion, with the pulsed laser ablation method and the AJ printing techniques, arbitrarily structural design and proportional QD addition make the developed QD white-light LEDs benefit for the future applications both in display and lighting industries.

## Methods

### Femtosecond laser

An 800 nm Ti:Sapphire femtosecond laser (Clark-MXR, CPA-2010) was used with the following parameters: single pulse energy: 20 μJ, frequency: 1000 Hz, and pulse duration 100 *fs*. The number of laser pulses was varied from 1 to 1000 at a single spot. The sample surface was irradiated at normal incidence by a focused linearly polarized laser beam.

### Aerosol jet spray coating

There are two special designs for the spraying mechanism. One is the air-injection in the nozzle and intermittent spraying frequency of 5–10 Hz and the other is based on constant stirring system.

### EL characterizations

The light intensity can be computed by integrating the light intensity in entire volume of a sphere. The power supplies (Keithley 2400) can supply different currents to the sample and the spectrometer can analyze the intensity of each wavelength in the sphere.

## Additional Information

**How to cite this article**: Wang, S.-W. *et al*. Pulsed-laser micropatterned quantum-dot array for white light source. *Sci. Rep.* **6**, 23563; doi: 10.1038/srep23563 (2016).

## Supplementary Material

Supplementary Information

## Figures and Tables

**Table 1:** White light emission performance of the developed QD-based white-light LEDs with different RGB QD ratios.

	Case1	Case2	Case3	Case4
B:G:R	3:4:4	10:4:3	10:4:5	10:4:10
(x, y)	(0.325, 0.395)	(0.271, 0.312)	(0.302, 0.311)	(0.363, 0.307)
CCT	5752.6 K	9705 K	7334 K	3936 K
