# Supplementary material for: Pulsed-laser micropatterned quantum-dot array for white light source
Source: Sci Rep. 2016 Mar 23;6:23563. doi: 10.1038/srep23563 (PMC4804257; doi:10.1038/srep23563)
Supplement: Supplementary Information [file srep23563-s1.pdf]

Supplementary Information

Pulsed-laser micropatterned quantum-dot array for white light source

Sheng-Wen Wang[1], Huang-Yu Lin[1], Chien-Chung Lin[2], Tsung Sheng Kao[1,†] Kuo-Ju Chen[1], Hau-Vei Han[1], Jie-Ru Li[1], Po-Tsung Lee[1], Huang-Ming Chen[1], Ming-Hui Hong[3] and Hao-Chung Kuo[1,*]

[1]Department of Photonics & Institute of Electro-Optical Engineering, National Chiao Tung University, Hsinchu 30010, Taiwan

[2]Institute of Photonic System, National Chiao Tung University, Tainan 711, Taiwan

[3]Department of Electrical and Computer Engineering, National University of Singapore, 4 Engineering Drive 3, 117576 Singapore, Singapore

[†]tskao@nctu.edu.tw; [*]hckuo@faculty.nctu.edu.tw

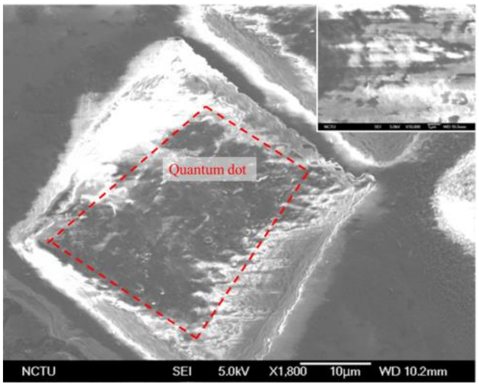

Supplementary Figure S1. SEM image of microstructure with QDs droplets.

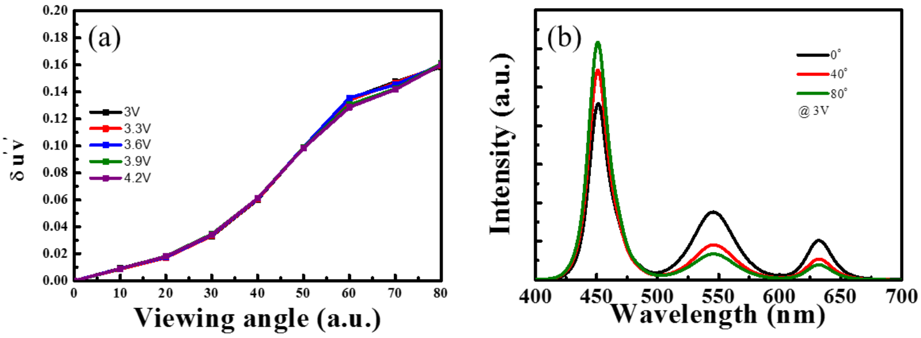

Supplementary Figure S2. (a) Color shift of the PL QDs LED at different driving voltages, and (b) the normalized EL spectra of the PL QDs LED at different viewing angles.

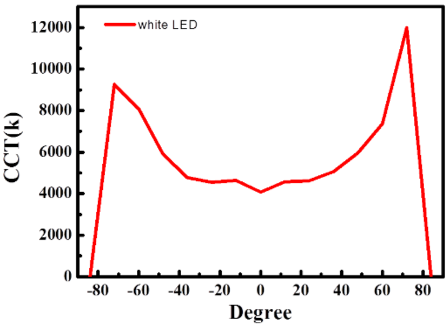

Supplementary Figure S3. Simulation of angle-dependent CCT for the blue flip chip with the emitting layer covered to generate the white LED.

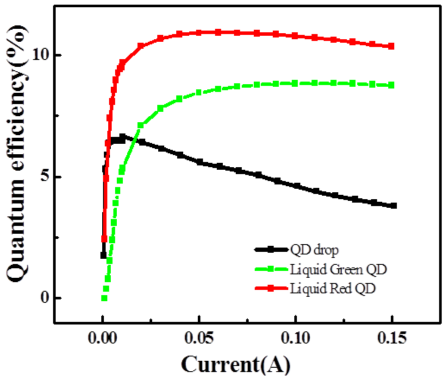

Supplementary Figure S4. Quantum efficiency as a function of current from 1 mA to 150mA

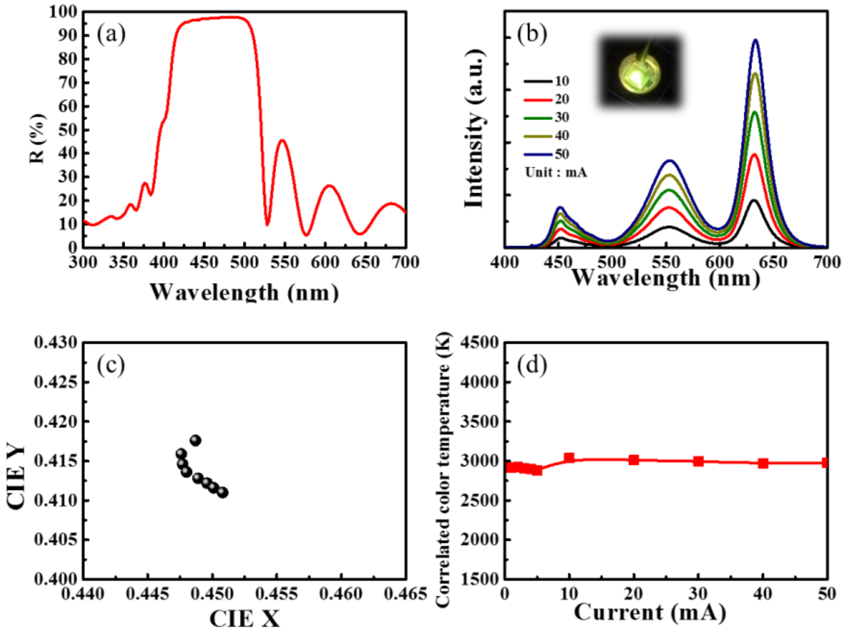

Supplementary Figure S5. Electroluminescence characterization of QDs LED with blue DBR. (a) Reflectance spectrum of the blue DBR structure. (b) Relative emission spectra of the QDs LEDs with DBR operated at the driving current in a range from 10 mA to 50 mA. The inset optical microscope image shows the light emission from the WLED device. (c) The trace of light illumination from the QDs WLEDs in CIE1931 chromaticity diagram. (d) The CCT values of the WLED operated at different driving currents.
